# A Marine Anthraquinone SZ-685C Overrides Adriamycin-Resistance in Breast Cancer Cells through Suppressing Akt Signaling

**DOI:** 10.3390/md10040694

**Published:** 2012-03-23

**Authors:** Xun Zhu, Zhenjian He, Jueheng Wu, Jie Yuan, Weitao Wen, Yiwen Hu, Yi Jiang, Cuiji Lin, Qianhui Zhang, Min Lin, Henan Zhang, Wan Yang, Hong Chen, Lili Zhong, Zhigang She, Shengping Chen, Yongcheng Lin, Mengfeng Li

**Affiliations:** 1 Key Laboratory of Tropical Disease Control (Sun Yat-sen University), Ministry of Education, Guangzhou 510080, China; Email: zhuxun8@mail.sysu.edu.cn (X.Z.); hzj100@163.com (Z.H.); wujh@mail.sysu.edu.cn (J.W.); yuanjie@mail.sysu.edu.cn (J.Y.); wwt11@163.com (W.W.); yiwen_810@126.com (Y.H.); cherie1011@126.com (C.L.); 762983161@qq.com (Q.Z.); xiayutian0302@163.com (M.L.); 872824573@qq.com (H.Z.); cindy364@163.com (W.Y.); chenshp@mail.sysu.edu.cn (S.C.); 2 Guangdong Province Key Laboratory of Functional Molecules in Oceanic Microorganism (Sun Yat-sen University), Bureau of Education, Guangzhou 510080, China; Email: chenhong_212@yahoo.com.cn (H.C.); zhonglili42@yahoo.com.cn (L.Z.); cesshzhg@mail.sysu.edu.cn (Z.S.); ceslyc@mail.sysu.edu.cn (Y.L.); 3 Department of Microbiology, Zhongshan School of Medicine, Sun Yat-sen University, Guangzhou 510080, China; 4 Department of Biochemistry, Zhongshan School of Medicine, Sun Yat-sen University, Guangzhou 510080, China; 5 Department of Cardiology, The First Affiliated Hospital of Sun Yat-Sen University, 58 Zhongshan Road II, Guangzhou 510080, China; Email: jiangyi77@163.com; 6 Department of Cardiology, The First People’s Hospital of Zigong, 42 Shangyihao Road I, Zigong 643000, China; 7 School of Chemistry and Chemical Engineering, Sun Yat-sen University, Guangzhou 510275, China

**Keywords:** SZ-685C, breast cancer, chemoresistance, Akt

## Abstract

Breast cancer remains a major health problem worldwide. While chemotherapy represents an important therapeutic modality against breast cancer, limitations in the clinical use of chemotherapy remain formidable because of chemoresistance. The HER2/PI-3K/Akt pathway has been demonstrated to play a causal role in conferring a broad chemoresistance in breast cancer cells and thus justified to be a target for enhancing the effects of anti-breast cancer chemotherapies, such as adriamycin (ADR). Agents that can either enhance the effects of chemotherapeutics or overcome chemoresistance are urgently needed for the treatment of breast cancer. In this context, SZ-685C, an agent that has been previously shown, as such, to suppress Akt signaling, is expected to increase the efficacy of chemotherapy. Our current study investigated whether SZ-685C can override chemoresistance through inhibiting Akt signaling in human breast cancer cells. ADR-resistant cells derived from human breast cancer cell lines MCF-7, MCF-7/ADR and MCF-7/Akt, were used as models to test the effects of SZ-685C. We found that SZ-685C suppressed the Akt pathway and induced apoptosis in MCF-7/ADR and MCF-7/Akt cells that are resistant to ADR treatment, leading to antitumor effects both* in vitro* and *in vivo.* Our data suggest that use of SZ-685C might represent a potentially promising approach to the treatment of ADR-resistant breast cancer.

## 1. Introduction

Chemotherapy remains an important and irreplaceable modality of cancer treatment that utilizes chemical drugs to kill, or inhibit, the growth of cancer cells. To date, hundreds of compounds have been used in cancer chemotherapy [1]. Employment of chemotherapy in patients with cancer, however, often fails to achieve clinical benefit due to severe adverse effects. Furthermore, development of resistant to chemotherapeutic drugs, particularly through multidrug resistance (MDR), represents a major impediment to successful chemotherapy [2]. Tumor cells are found to adopt multiple mechanisms to resist drugs, including decreased uptake of drugs and/or enhanced efflux of drugs, altered metabolism of drugs, alterations in drug targets, activation of the detoxification system, enhanced DNA repair ability and inhibition of apoptosis [2]. Hence, an increasing demand for the availability of new lead compounds drives an intense search for new bioactive natural products. Currently, it is of particular note that pharmacologically-active compounds derived from marine organisms represent a promising source for developing new investigational anti-cancer drugs, as they produce a wide variety of metabolites that are structurally unique and biologically active [3].

Breast cancer remains the most commonly diagnosed type of cancer among women and a major health problem worldwide [1]. Despite many advances in the treatment of breast cancer, resistance to anti-breast cancer agents represents a major challenge to the development of successful treatment. Mechanistically, resistance to chemotoxic agents in breast cancer patients is most often associated with the action of ATP-binding cassette (ABC) drug transporters, which include ABC transporter-subfamily B member 1 (ABCB1, or P-glycoprotein/P-gp), subfamily C member 1 (ABCC1, or multidrug resistance-associated protein 1/MRP1) and subfamily G member 2 (ABCG2, or breast cancer resistance protein/BCRP) [2,4].

Several new studies have shown that growth factor receptor-mediated signal transduction, such as the HER2/phosphoinositide-3-kinases(PI3K)/Akt signaling pathway, has been implicated in conferring resistance to conventional chemotherapy against breast cancer [5,6,7,8]. Moreover, the PI3K/Akt pathway has been implicated in the regulation of a wide variety of cellular processes including survival, proliferation, growth and metabolism [9]. Genetic deregulation of PI3K activity has been found in a wide variety of tumor types, including somatic oncogenic gain-of-function mutations and amplification of genes encoding key components along the pathway [10,11]. Several new studies suggest that HER2/PI3K/Akt signaling may confer resistance to a panel of chemotherapeutic agents with different mechanisms of actions: adriamycin (an anthracycline) [6,7,8], mitoxantrone (an anthracycline) [8], 5-fluorourocil (an antimetabolite) [7,8], etoposide (a DNA-damaging agent) [7,8], camptothecin (a topoisomerase I inhibitor) [7] *etc*. Such resistance may be relevant to the effects of MDR in breast cancer. It has been documented that activation of Akt by HER2/PI-3K plays an important role in conferring broad-spectrum chemoresistance in breast cancer cells. Thus, Akt is believed to be a novel molecular target for therapies that would improve the outcome of anti-breast cancer chemotherapy in patients with breast cancer [12].

Anthracyclines are the most commonly used anti-cancer treatment. Anthracycline drugs, such as Epirubicin and Mitoxantrone, are still in the first-line treatment against many types of cancer, including acute leukemia, Hodgkin’s lymphoma and breast cancer [13,14]. Clinical application of anthracyclines, however, is limited by their tendency to induce multidrug resistance [14]. It is therefore important to identify new anthracenedione derivatives with improved pharmacological and toxicological profiles. In our previous study, SZ-685C [15], a secondary metabolite purified from the mangrove endophytic fungus No. 1403, collected from the South China Sea, was found to exhibit structural similarity to anthracyclines, the anti-cancer drug widely used in the clinic. We demonstrated that SZ-685C induces apoptosis through Akt signaling, which consequently led to antitumor effects both* in vitro* and *in vivo*, suggesting that SZ-685C may be a potentially promising Akt inhibitor and anti-cancer drug candidate [15]. 

The current study attempts to address whether SZ-685C can reverse chemoresistance by suppressing the Akt signaling in human breast cancer cells. Our results demonstrated that SZ-685C suppressed the proliferation of ADR-resistant MCF-7/ADR and MCF-7/Akt breast cancer cells as well as the growth of MCF-7/ADR xenografts, suggesting a potentially promising approach to the treatment of ADR-resistant breast cancer.

## 2. Results

### 2.1. Growth Inhibition of ADR-Resistant Human Breast Cancer Cell Lines Induced by SZ-685C

**Figure 1:**
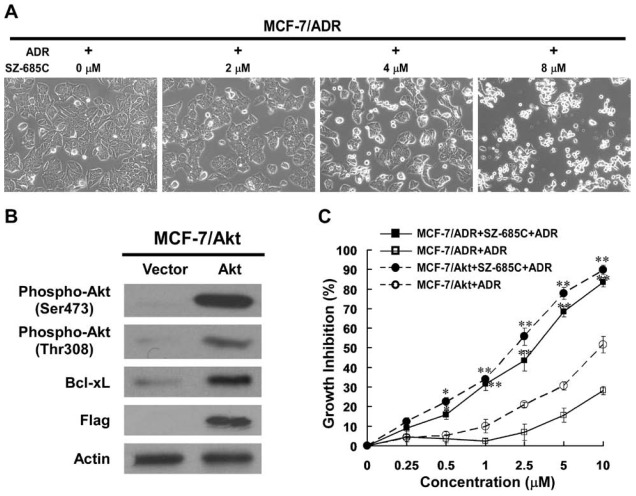
Effect of SZ-685C on the growth of MCF-7/ADR and MCF-7/Akt cells. Cells were seeded in 96-well plates and incubated with different concentrations of SZ-685C or ADR as noted for 48 h at 37 °C. (**A**) Effect of SZ-685C on morphology of MCF-7/ADR (200×); (**B**) MCF-7/Akt cells stably expressing Akt were assessed for changes in phosphorylation status of Akt at Thr308 and Ser473 and total levels of Flag-Akt protein; (**C**) Viabilities were determined by MTS assay. Data points are presented as means ± SD of triplicated experiments. The Student’s *t* test was performed to compare the growth of MCF-7/ADR and MCF-7/Akt cells (* indicates *p *< 0.05, and ** indicates *p *< 0.01).

To investigate whether SZ-685C was able to inhibit the growth of ADR-resistant human breast cancer cells, its effects on the MCF-7/ADR, a cell line with ADR resistance derived from the parental MCF-7 breast cancer cells, was examined using MTS assay. Upon treatment of SZ-685C for 48 h, cultured MCF-7/ADR exhibited markedly inhibited growth, as compared with vehicle-controlled cells in a dose-dependent manner (Figure 1, A-C). Calculated IC_50_s, *i.e.*, concentrations of SZ-685C required for decreasing the growth rate of the cells by 50%, were 4.17 μM for MCF-7/ADR (Table 1) and 7.38 μM for MCF-7, indicating decreasing the ADR resistance factor (RF) from 19.19 to 0.57. The effect of SZ-685C on the viability of ADR-resistant cell lines of other two types of human cancer, including K562/ADR (human erythromyeloid leukemia) and HL-60/ADR (human promyelocytic leukemia) were further evaluated. The results presented in Table 1 reveal significant inhibitory effects of SZ-685C on both tested cell lines, with IC_50 _values of 1.35 μM for K562/ADR and 1.76 μM for HL-60/ADR, and decreasing the ADR resistance factor from 58.33 to 1.24, and 54.94 to 0.91 by SZ-685C, respectively, after 48 h of treatment (Table 1).

**Table 1:** IC_50_ values of SZ-685C on various ADR-resistant cancer cell lines.

Cell Lines	ADR		SZ-685C	
	IC_50_	RF	IC_50_	RF
MCF-7	0.96	-	7.38	-
MCF-7/ADR	18.42	19.19	4.17	0.57
MCF-7/Akt	7.69	8.01	3.36	0.46
K562	0.15	-	1.09	-
K562/ADR	8.75	58.33	1.35	1.24
HL-60	0.33	-	1.94	-
HL-60/ADR	18.13	54.94	1.76	0.91

We also constructed a Flag-tagged constitutively active (PI3K-independent) Akt expression vector, termed Flag-DPH-Akt1-farn, by replacing the phospholipid-interactive PH domain of Akt1 with a farnesylation sequence to direct constitutive membrane anchoring, as previously described [7]. Using this vector, retroviral activity was generated in a modified MCF-7 cell line stably overexpressed Flag-DPH-Akt1-farn (MCF-7/Akt1), as well as a control pMSCV backbone vector (MCF-7/Vector). As shown in Figure 1B, the results of western blotting analysis of the control-vector transfected clone MCF-7/Vector and a representative Flag-DPH-Akt1-farn vector-transduced clone (MCF-7/Akt1) demonstrated that expression of high-level farnesylated Flag-DPH-Akt1-farn, but not endogenous Akt, a markedly increased phosphorylation of Akt1. In parallel with the observed increase of Akt phosphorylation, Bcl-xL, a crucial anti-apoptotic effector downstream to Akt signaling, was upregulated in MCF-7/Akt cells. Further analysis summarized in Table 1 suggested that the constitutively phosphorylated Akt conferred MCF-7 cell resistance to ADR, and, as shown in Figure 1C SZ-685C, and exhibited a marked inhibition on the survival of MCF-7/Akt cells in a dose-dependent manner. In addition, the IC_50_ value was 3.36 μM for MCF-7/Akt, decreasing the ADR resistance factor from 8.01 to 0.46.

### 2.2. SZ-685C Induced Apoptosis in MCF-7/ADR and MCF-7/Akt Cells

**Figure 2:**
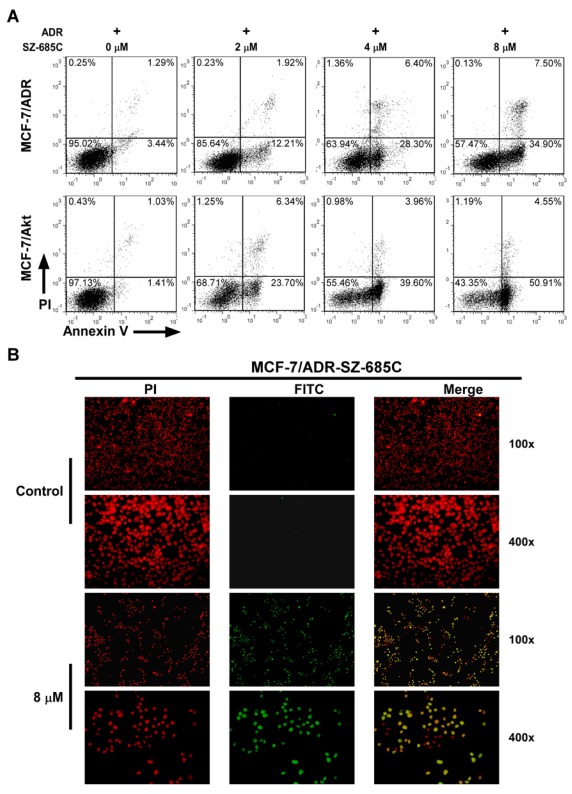
Induction of apoptosis in MCF-7/ADR and MCF-7/Akt cells by SZ-685C. (**A**) AnnexinV-FITC/PI staining of MCF-7/ADR and MCF-7/Akt cells treated with SZ-685C. Cells were exposed to different concentrations (0, 2, 4, 8 μM) of SZ-685C and 1 μM ADR for 12 h. Cells were collected and subjected to Annexin V-FITC/PI staining and analyzed by flow cytometry. (**B**) TUNEL assay of MCF-7/ADR cells treated with SZ-685C. Cells were treated with 8 μM SZ-685C as noted for 24 h and labeled with fluorescein-12-dUTP (red) and PI counterstaining (green).

Apoptosis assays exhibited that upon treatment of SZ-685C for 12 h at 2, 4, 8 μM, respectively, numbers of apoptotic MCF-7/ADR and MCF-7/Akt cells (Annexin V^+^/PI^−^), as revealed by Annexin-V binding, markedly increased in a dose-dependent manner (Figure 2A) When the Terminal deoxynucleotidyl transferase-mediated dUTP nick end labeling (TUNEL) assay was performed to assess DNA fragmentation, as a late event in the process of apoptosis of MCF-7/ADR cells, a higher amount of TUNEL-positive cells were visualized in MCF-7/ADR cells treated for 24 h with SZ-685C at 8 μM, as compared to the control (Figure 2B). The results of two apoptosis assays, *i.e.*, the Annexin V-binding and TUNEL assays, strongly suggested a pro-apoptotic effect of SZ-685C on ADR-resistant MCF-7 cells.

### 2.3. Activation of Both the Extrinsic and Intrinsic Apoptotic Pathways by SZ-685C

**Figure 3:**
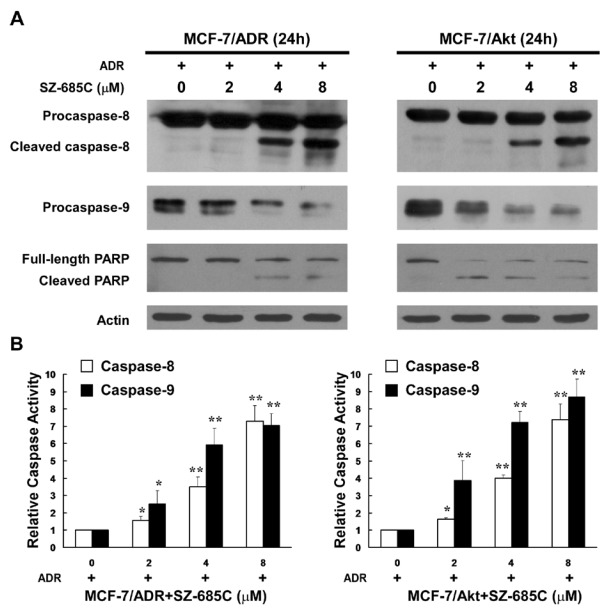
Activation of caspases and PARP in MCF-7/ADR and MCF-7/Akt cells by SZ-685C. (**A**) Western blotting analysis of caspases and PARP in MCF-7/ADR and MCF-7/Akt cells after SZ-685C treatment at different concentrations for 48 h using antibodies against caspase-8, -9, and PARP. Actin was used as an internal control; (**B**) Enzymatic activities of caspases in SZ-685C treated MCF-7/ADR and MCF-7/Akt cells, as assessed by colorimetric assay. The fold-increases in the activities of caspases-8 and -9 were determined by comparison with those of the treated control cells. Results are presented as means ± SD. The asterisks indicate statistically significant differences as compared with the control: *p < 0.05, **p < 0.01.

Recent evidence suggests that apoptotic mechanisms mediate drug-induced cell death, and that the activation of effector caspases plays a central role in the execution of apoptosis [16]. Therefore, to estimate the cytocidal potential of chemotherapeutic agents, it is important to quantify their abilities to induce apoptosis and activate caspases. To further characterize the cell apoptotic process in MCF-7/ADR and MCF-7/Akt cells, pro-apoptotic caspases, *i.e.*, caspase-9 and -8, and the effector molecule PARP, were examined on the two cell lines treated or untreated, comparatively, with SZ-685C. Our results (Figure 3A) displayed that treatments with various concentrations of SZ-685C for 24 h dramatically increased activating cleavage of caspase-8 dose-dependently in MCF-7/ADR and MCF-7/Akt cells. In consistence with the results of western blotting analysis, the enzymatic activity of caspase-8 in an enzymatic assay showed a dose-dependent increase with SZ-685C treatment (Figure 3B). Concurrently, cleavage of the caspase-9 precursor and increased caspase-9 activity were also detected (Figure 3A,B). Cleavage of PARP from 116 kDa to 85kDa was clearly demonstrated after SZ-685C treatment both in MCF-7/ADR and MCF-7/Akt cells (Figure 3A). Taken together, these data suggest that apoptosis induced by SZ-685C in ADR-resistant breast cancer cells may involve both intrinsic and extrinsic pathways.

### 2.4. Inhibition of Akt Phosphorylation and Its Downstream Substrates by SZ-685C

**Figure 4:**
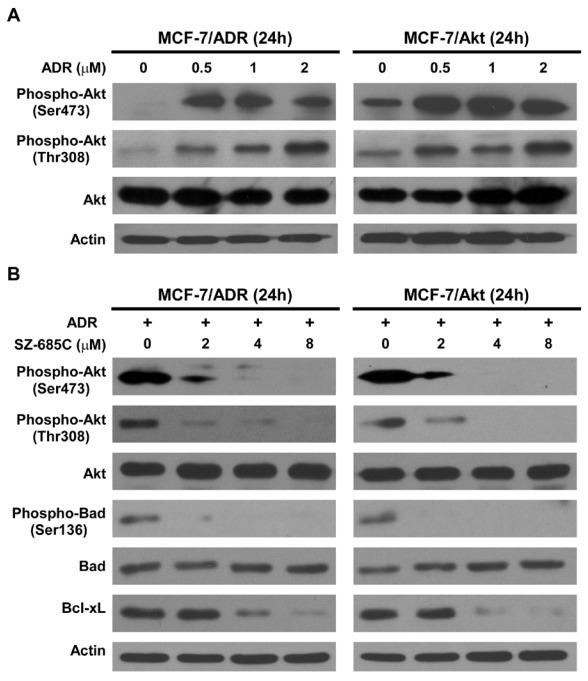
Effect of SZ-685C on the Akt signaling pathway in MCF-7/ADR and MCF-7/Akt cells. Cells were treated with different concentrations of ADR (**A**) and SZ-685C (**B**) for 48 h as noted. Western blotting analysis was performed using antibodies against phospho-Akt (Ser473 or Thr308), total Akt, phospho-Bad (Ser 136), total Bad, and Bcl-xL, with Actin used as a loading control.

In an effort to further understand the signaling cascade that mediates the pro-apoptotic effect of SZ-685C on MCF-7/ADR and MCF-7/Akt cells, changes in phosphorylation status of Akt were investigated. As shown in Figure 4, for both tested ADR-resistant breast cancer cell lines, in which Akt was constitutively activated upon treatment with ADR (Figure 4A). SZ-685C treatment resulted in a dose-dependent decrease of phospho-Ser473-Akt and phospho-Thr308-Akt without an effect on total Akt expression (Figure 4B). Bad is a pro-apoptotic member of the Bcl-2 family that promotes cell death by displacing Bax from binding to Bcl-2 and Bcl-xL. Phosphorylation at Ser112 and Ser136 promotes binding of Bad to 14-3-3 proteins to prevent an association between Bad with Bcl-2 and Bcl-xL. Akt can phosphorylate Bad at Ser136 to promote cell survival [17]. In parallel with the observed reduction of Akt phosphorylation, phosphorylation of Bad (Ser136), a crucial mediator downstream of Akt signaling, was also inhibited by SZ-685C treatment in a dose-dependent manner without an effect on total Bad expression (Figure 4B). Moreover, Bcl-xL, another anti-apoptotic effector downstream to Akt signaling, was downregulated in response to SZ-685C treatment in MCF-7/ADR and MCF-7/Akt cells (Figure 4B).

### 2.5. Anti-Tumor Effect of SZ-685C on ADR-Resistant Breast Cancer Xenografts *in Vivo*

**Figure 5:**
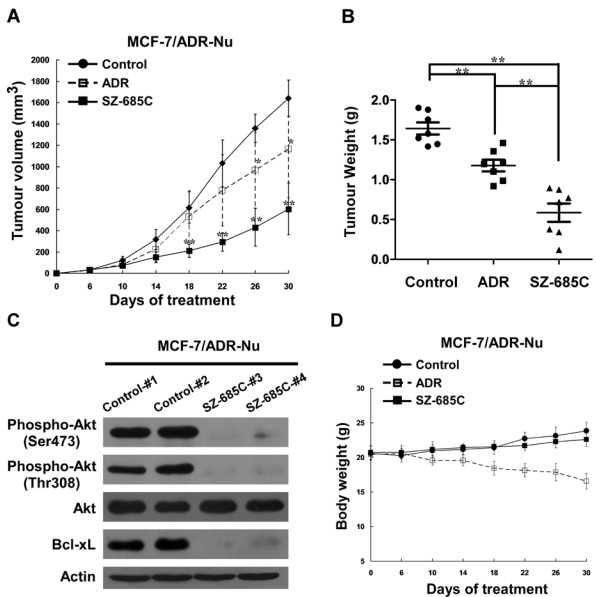
*In vivo* anti-tumor effect of SZ-685C on xenografted MCF-7/ADR tumors in nude mice. Viable MCF-7/ADR cells (1 × 10^7^ cells/mouse) were inoculated subcutaneously in the right mammary pads of female nude mice. After solid tumor formation, the mice received an intraperitoneal injection of SZ-685C (50 mg/kg), ADR (8 mg/kg) or vehicle-control (0.5% DMSO) every four days for about one month. (**A**) Time-response curve of the effect of SZ-685C on the growth of xenografted MCF-7/ADR tumor; (**B**) Inhibition ratio calculated, based on tumor tissues weight for each group; (**C**) Western blotting analysis was performed using antibodies against phospho-Akt (Ser473 or Thr308), total Akt, and Bcl-xL, with Actin used as a loading control, in two representative tumor tissues, followed by treated with SZ-685C; (**D**) Time-response curve of the effect of SZ-685C or ADR on the body weight of the xenografted mice. Results are presented as means ± SD. The asterisks indicate statistically significant differences as compared with the control, with * and ** indicating *p *< 0.05 and *p *< 0.01, respectively, as tested using one-way analysis of variance (ANOVA) followed by Dunnett’s multiple comparisons.

To determine the anticancer activity of the SZ-685C against ADR-resistant breast cancer *in vivo*, we next tested the therapeutic effect of SZ-685C on MCF-7/ADR xenograft in a nude mice model. When the tumor volumes were assessed on day 6 after inoculation, all the animals in the group had developed spinal cord tumors (7/7, or 100%), with a mean volume (±SD) of approximately 30 mm^3^. The growth of the xenograft tumors were monitored following injection with SZ-685C or ADR. A marked inhibition of the growth of the xenografted tumors formed by the MCF-7/ADR cells treated with SZ-685C was observed. After six SZ-685C injections, the volume of the xenografted breast tumor was significantly inhibited by 63.23% in SZ-685C-treated nude mice, as compared with the tumor-inhibitory rate, *i.e.*, 29.11% of ADR (Figure 5A). The weight of the xenografted tumor at experimental endpoint was significantly inhibited by 64.32% and 28.34%, respectively, in SZ-685C- and ADR-treated nude mice (Figure 5B). Furthermore, decreases of phospho-Ser473-Akt, phospho-Thr308-Akt and Bcl-xL, without an effect on total Akt expression, were observed in two representative tumor tissues, followed by treated with SZ-685C (Figure 5C). Meanwhile, no detectable toxic effects, such as signs of discomfort, change in animal behavior, or weight loss (Figure 5D), was found in SZ-685C-treated animals. In contrast, the ADR-treated animals exhibited a certain degree of drug toxicity, including significant weight loss (Figure 5D). These data strongly suggest that systemically-delivered SZ-685C was able to inhibit the growth of established ADR-resistant tumors *in vivo*.

## 3. Discussion

Anthracyclines, such as ADR, are a class of anti-cancer chemotherapeutic drugs commonly used in the clinic to treat various types of human cancer, including breast cancer. Clinical application of anthracyclines, however, is frequently impeded by their severe adverse side effects, e.g., cardiotoxicity [18,19], and myelosuppression [20], as well as the tendency to induce multidrug resistance [21]. There is, therefore, an urgent need to identify new anthracenedione derivatives with improved pharmacological and toxicological profiles. In our previous study that aimed at screening novel anticancer lead compounds from a metabolite library derived from marine microorganisms isolated from the South China Sea, SZ-685C was selected for its potent anticancer activity *in vitro* as well as *in vivo *[15]. We have demonstrated that SZ-685C selectively kills cancer cells via activating both caspase-8- and caspase-9-mediated apoptotic mechanisms by, at least in part, suppressing the phosphorylation of Akt. In our current study, we provide evidence that SZ-685C is able to kill breast cancer cells resistant to the anti-cancer effect of ADR, and thereby might represent a new, potentially promising approach to the treatment of anthrocycline-resistant breast cancer.

This study employed a cellular model MCF-7/ADR that was originally derived from the human breast cancer cell line MCF-7, and selected for resistance to ADR. It is of note that the parental cell line of the MCF-7/ADR and MCF-7/Akt models, *i.e.*, the MCF-7 cell line, is relatively well differentiated and estrogen-dependent, containing receptors for estrogen and progesterone [22,23]. Interestingly, these estrogen responses are lost in MCF-7/ADR cells. It has been reported that the MCF-7/ADR cells form tumors in the nude mice in the absence of exogenous estrogen administration, while the formation of tumors in nude mice by wild-type MCF-7 cells is estrogen-dependent [24]. Furthermore, MDR presented by MCF-7/ADR cells is also associated with a loss of mitogenic response to estrogen and the development of cross-resistance to the anti-estrogen, 4-hydroxytamoxifen. These changes in the hormonal sensitivity and estrogen-independent tumorigenicity of the multidrug-resistant MCF-7/ADR cell line might be attributable to loss of estrogen receptor and a concomitant increase in the level of other receptors, such as those for epidermal growth factor. The results obtained from our current study warrants further exploration of the possibility of SZ-685C to sensitize breast cancer cells to anti-estrogen drugs.

A second test model used in our study,* i.e.*, the MCF-7/Akt cell, is a derivative of the MCF-7 cells, engineered to express constitutively activated Akt. As a central mediator of PI3K signaling, Akt plays a key role in protecting cells from various apoptotic stimuli by phosphorylating diverse downstream targets, such as Forkhead family transcription factors, Bad, Bcl-xL, InB kinase, MDM2 and procaspase-9 [9,25]. Akt also interacts, directly or indirectly, with numerous other regulatory proteins, including cyclin D, p21^Cip1^, glycogen synthase kinase (GSK)-3, and mTOR [9,25]. The PI3K/Akt pathway has thus been implicated in the regulation of a wide variety of cellular processes, including survival, proliferation, growth, and metabolism. Under physiological conditions, PI3Ks were localized to the plasma membrane upon activation, where it phosphorylates the substrate phosphatidylinositol-4, 5-diphosphate (PIP2), integral components of the plasma membrane, to phosphatidylinositol-3, 4, 5-triphosphate (PIP3) [26]. PIP3 functions as a membrane-bound second messenger by recruiting a subset of pleckstrin homology (PH), domain-containing proteins, such as Akt and 3-phosphoinositide-dependent kinase 1 (PDK1), to the membrane, in which they become activated and initiate downstream signaling events [26].

It was previously reported that MCF-7 cells expressing a constitutively active Akt, in which the phospholipid-interactive PH domain of Akt was replaced by a farnesylation sequence for constitutive membrane anchorage, showed a broad-spectrum chemoresistance on breast cancer cells [6,7]. Here, we also generated MCF-7/Akt stable cell lines by using the same approach, in which Akt protein was cleavable by the farnesyl-esterases that were expressed in mammalian cells, thereby allowing its cytoplasmic relocalization. Our results obtained from the current study indicate that the Akt kinase in MCF-7/Akt was constitutively active, as expected, and was sufficient to mediate resistance to ADR. The results are in line with the observation that the modified MCF-7 cells expressing constitutively activated Akt or a high level of HER2 were resistant to a panel of chemotherapeutic agents [6,7,8]. These results strongly support the hypothesis that deregulated over-activation of Akt plays an important role in resistance to chemotherapy. SZ-685C, a novel anthracycline compound, as evidenced in this study, is able to potently suppress the phosphorylation of Akt and subsequently induce apoptosis in ADR-resistant breast cancer cells in which Akt kinase is over-activated. The involvement of Akt in human cancer oncogenesis and chemoresistance indicates that Akt is an important target for screening anti-cancer drugs against various types of cancer cells including those resistant to conventional chemotherapy.

## 4. Experimental Section

### 4.1. Chemicals

Unless otherwise stated, laboratory chemicals, bacterial culture reagents, antibiotics and disposable labware were purchased from the Sangon Biotech (Shanghai, China), Sigma-Aldrich (St. Louis, MO, USA), or BBI (Gaithersburg, MD, USA). SZ-685C was prepared and purified from mangrove endophytic fungus No. 1403 as previously reported [15], and its structure was identified by interpretation of spectral data (MS, ^1^H NMR, ^13^C NMR, 2D NMR) and X-ray single crystal diffractive technique. The compound was dissolved in 0.5% dimethylsulfoxide (DMSO) at a concentration of 1 mM as stock solution, and diluted according to experimental requirements when used. ADR was purchased from Merck (Merck KGaA, Darmstadt, Germany).

### 4.2. Cell Culture

MCF-7/ADR (Human breast cancer), K562/ADR (Human erythromyeloid leukemia), and HL-60/ADR cells (Human promyelocytic leukemia) were obtained from Keygen Biotech (China). Cells were grown in Dulbecco’s Modified Eagle Medium (DMEM) (Gibco-Invitrogen, Carlsbad, CA, USA), supplemented with 10% fetal bovine serum (Hyclone, Logan, UT), 2 mM L-glutamine, 100 μg/mL streptomycin and 100 Units/mL penicillin (Gibco-Invitrogen, Carlsbad, CA), 1 μM ADR (to maintain ADR resistance phenotype, upon treatment with or without SZ-685C). All of the cells used in this study were maintained at 37 °C in a humidified atmosphere, 5% CO_2_ incubator.

### 4.3. Establishment of Stable Cell Lines Expressing Constitutively Activated Akt

Flag-tagged constitutively activated (PI3K-independent) Akt expression vector was constructed by replacing the phospholipid-interactive PH domain of Akt1 with a farnesylation sequence for directing constitutive membrane anchoring, termed Flag-DPH-Akt1-farn vector, as previously described [7]. The final PCR product encoded Akt1 that lacks the PH domain (aminoacids 1-106) with an *N*-terminal fusion of the Flag epitope (DYKDDDDK) and a *C*-terminal fusion of a poly-basic tail and a farnesylation sequence (KKKKKKSKTKCVIM). The PCR fragment was subcloned into the pMSCV-Puro vector (Invitrogen, Carlsbad, CA, USA) in the restriction sites Bgl II and EcoR I. The final Akt sequence was verified by nucleotide sequencing analysis. Recombinant retroviruses were prepared by transfecting 2 μg of each of the pMSCV-Flag-DPH-Akt1-farn plasmid DNAs and pMSCV-Puro vector into the packaging cells 293FT, together with 2 μg of the pIK plasmid. These viruses were used to infect MCF-7 cells, and stably transduced cell lines were selected in DMEM medium supplemented with puromycin (5 μg/mL). To verify the expression of the Flag-DPH-Akt1-farn proteins, cell lysates were collected and examined using Western blotting analysis.

### 4.4. Cell Viability Analysis

Cells (1 × 10^4^ cells/well) in growth medium were seeded in 96-well flat-bottom plates (in triplicates) for 24 h in the absence or presence of various concentrations of SZ-685C and 1 μM ADR for additional 48 h. Cell viability was measured by using the MTS (3-(4,5-dimethylthiazol-2-yl)-5-(3-carboxymethoxyphenyl)-2-(4-sulfophenyl)-2*H*-tetrazolium) assay to monitor cell proliferation, according to the manufacturer’s recommendations. Briefly, 20 μL MTS solution (CellTiter 96Aqueous One Solution reagent, Promega, Madison, WI, USA) were added to each well and incubated for an additional 4 h at 37 °C. The absorbance was measured at 490 nm using a microplate reader (Bio-Tek Synergy 2, Winooski, VT, USA). The control group was defined as cells that were treated with 1 μM ADR and not exposed to SZ-685C. Cell growth inhibition was determined using the following formula according to a previously published method: growth inhibition (%) = (1 − O.D. of treated cells/O.D. of control cells) × 100% [15]. The half maximal inhibitory concentration (IC_50_) was calculated with Bliss’s software and the data were analyzed by SPSS [15]. The resistance factor (RF) was calculated as the ratio of the IC_50_ for the resistant cell line to the IC_50_ for the sensitive cell line. All experiments were performed three times from which mean values were calculated.

### 4.5. Annexin V-FITC/Propidium Iodide (PI) Staining Assay

The Annexin V/Propidium iodide (PI) dual staining assay was employed to detect viable and early apoptotic cells. Cells (2 × 10^5^ cells/well) were seeded in 60 mm plates and allowed to settle for 24 h before treatment with various concentrations (0, 2, 4, 8 μM) of SZ-685C and 1 μM ADR for an additional 12 h. The cells were trypsinized after washing for three times with PBS, and incubated in 500 μL binding buffer containing annexin V-FITC and PI in the dark for 10 min at room temperature, according to the manufacturer’s instructions (Keygen Biotech, China), followed by measurement with flow cytometric (FCM) analysis (FCAScan; Becton-Dickinson Immunocytochemistry Systems, San Jose, CA, USA). The percentage of apoptotic cells was defined by their distribution in a fluorescence dot plot using WinMdi 2.8 software [27].

### 4.6. Terminal Deoxynucleotidyl Transferase-Mediated dUTP Nick End Labeling (TUNEL) Assay

Apoptotic DNA fragmentation induced by SZ-685C was examined using the *in situ* DeadEnd^TM^ Fluorometric TUNEL System assay kit (Promega, Madison, WI, USA) according to the manufacturer’s protocol. Briefly, cells were plated in 24-well flat-bottom plates at a density of 1 × 10^5^ cells/well and treated with 8 μM SZ-685C and 1 μM ADR, or 1 μM ADR alone (Control group) for 24 h. Following SZ-685C treatment, cells were fixed in 4% paraformaldehyde at 4 °C for 30 min. Fixed cells were then permeabilized in 0.1% Triton X-100, and labeled with fluorescein-12-dUTP using terminal deoxynucleotidyl-transferase. After rinsing with PBS twice, the nuclei of cells were double-stained with PI (1 μg/mL) for 15 min. The localized green fluorescene of apoptotic cells (fluorescein-12-dUTP) was detected by fluorescence microscopy (Zeiss Axiovert100M, Carl Zeiss, Germany).

### 4.7. Caspase Activity Assay

Activity of Caspase-8 and Caspase-9 was measured using a caspase colorimetric assay kit (Keygen Biotech, China), according the manufacturer’s protocol. Briefly, following treatment of SZ-685C at different concentrations and 1 μM ADR for 48 h, cells were harvested and washed with PBS for two times, then resuspended in chilled 1 × lysis buffer. Cells lysates were centrifuged for 1 min at 10,000 × g after incubation on ice for 60 min. The supernatant was collected in a fresh tube, and the total protein concentration was determined by the Bradford protein assay kit (Keygen Biotech, China), according to the manufacturer’s protocol. Subsequently, 150 μg of each sample was diluted with 50 μL lysis buffer and added to 50 μL of 2 × reaction buffer containing 10 mM DTT in a 96-well plate. Then, 5 μL of a colorigenic substrate, IETD-*p*NA or LEHD-*p*NA, was added to each well, and the plate was incubated at 37 °C in the dark for 4 h. Absorbance at 405 nm was determined using a microplate reader (Bio-Tek Synergy 2, Winooski, VT).

### 4.8. Western Blotting Analysis

After treatment with SZ-685C or ADR at different concentrations for 48 h, floating cells and adherent cells were collected and lysed in 1× sample buffer containing 50 mM Tris-HCl (pH 7.4), 1 mM PMSF, 10% glycerol, 6% SDS, 5% mercaptoethanol and 0.1% bromophenol blue before sonication. The protein concentration of the extracts was determined by the Bicinchoninic Acid Protein Assay Kit (Thermo Fisher Scientific, Rockford, IL), according to the manufacturer’s instructions with BSA as the standard. The total cell lysate (40 μg of protein) was subjected to SDS-PAGE, and then transferred to PVDF membranes. After blocking with blocking buffer (Tris-buffered saline, TBS, containing 5% non-fat milk) for 1 h at room temperature, the membranes were incubated overnight at 4 °C with specific primary antibodies, namely: mouse anti-human caspase-8, mouse anti-human caspase-9 (BD Biosciences, San Jose, CA, USA); rabbit anti-human PARP, rabbit anti-human phospho-Akt (Ser473), rabbit anti-human phospho-Akt (Thr 308), rabbit anti-human Akt, Phospho-Bad (Ser136) antibody, Bad antibody, Bcl-xL Antibody (Cell Signaling, Beverly, MA, USA), and monoclonal anti-Actin antibody (Sigma-Aldrich, St. Louis, MO, USA). Further incubation with appropriate horseradish peroxidase (HRP)-conjugated secondary antibodies, depending on the primary antibody used, was performed for 1 h at room temperature. Immuno-reactive bands were detected using enhanced chemiluminescence kit (Thermo Fisher Scientific, Rockford, IL, USA) with Kodak film. Actin was used as a loading control for quantity normalization.

### 4.9. Xenografted Tumor Model and Anti-Tumor Effect of SZ-685C *in Vivo*

Female BALB/c-nu mice (18-20 g) were purchased from the Shanghai Laboratory Animal Center, Chinese Academy Sciences (Shanghai SLAC Laboratory Animal Co. LTD.), and were housed in barrier facilities on a 12 h light/dark cycle. Three days before implanting MCF-7/ADR cells, mice received a 0.8 mg β-estradiol pellet (Innovative Research, Toledo, OH, USA) subcutaneously in their front-back area. On day zero, MCF-7/ADR cells (1 × 10^7^ cells in 0.1 mL/mouse) were inoculated subcutaneously in the right mammary pad. Mice were ear-tagged and randomly divided into two treatment groups and a control group in a blinded fashion before treatment. On day six, formed tumors were measured, and the two treatment groups of animals received an intraperitoneal (i.p.) dosage of 200 μL SZ-685C (50 mg/kg body weight) and ADR (8 mg/kg body weight), respectively, every four days, while the vehicle-control animals received daily i.p. doses of 200 μL 0.5% DMSO solution per mouse. Tumors were measured every three days in a blinded manner by measuring perpendicular diameters with a digital caliper, and tumor volumes (mm^3^) were calculated using the following formula: volume = width × width × length × π/6. Data were presented as the means ± standard deviation (SD) of seven mice in each group. At the endpoint of the experiment, all the animals were euthanized, and the tumors were dissected and weighed. All experimental procedures were approved by the Institutional Animal Care and Use Committee of Sun Yat-sen University.

### 4.10. Statistical Analysis

The data given in the text are expressed as means ± SD. For MTS assay, statistical analyses were undertaken using the Student’s *t* test. Statistical significances of the differences in tumor volumes between treatment and control groups were determined by one-way analysis of variance (ANOVA) followed by Dunnett’s multiple comparisons.

## 5. Conclusions

In conclusion, our study provides the evidence that SZ-685C is a potent apoptosis inducer in breast cancer cells resistant to conventional ADR treatments *in vitro* as well as *in vivo*, and that such effects may be through suppressing Akt signaling, supporting the potential usefulness of combining SZ-685C with other therapeutic drugs in combating MDR in breast cancer chemotherapy.
